# Genetic variation and potential coinfection of *Wolbachia* among widespread Asian citrus psyllid (*Diaphorina citri* Kuwayama) populations

**DOI:** 10.1111/1744-7917.12566

**Published:** 2018-02-13

**Authors:** Chia‐Ching Chu, Mark Hoffmann, W. Evan Braswell, Kirsten S. Pelz‐Stelinski

**Affiliations:** ^1^ Department of Entomology and Nematology University of Florida Gainesville Florida USA; ^2^ Citrus Research and Education Center University of Florida Lake Alfred Florida USA; ^3^ Department of Horticultural Sciences North Carolina State University Raleigh North Carolina USA; ^4^ Center for Plant Health Science and Technology USDA APHIS Edinburg Texas USA

**Keywords:** bacteria, genetic diversity, Huanglongbing, plant disease, superinfection, vertically transmitted endosymbionts

## Abstract

*Wolbachia* can profoundly influence the survival, reproduction, and defenses of insect hosts. These interactions could potentially be harnessed for managing pests or insect‐transmitted diseases. *Diaphorina citri* Kuwayama is a phloem‐feeding pest capable of transmitting the putative causal agent of citrus greening, *Candidatus* Liberibacter asiaticus (*C*Las). Like many insects, *D. citri* is also infected with *Wolbachia* (*w*Di). Recent studies indicate that the relative abundance of *w*Di could be associated with the abundance of *C*Las, and that *w*Di may contribute to regulating expression of phage lytic cycle genes in *C*Las, suggesting the need for better understanding of *w*Di biology in general. This study investigated the genetic diversity of *w*Di among *D. citri* in populations spanning eleven countries and two U.S. territories. Six *Wolbachia* genes, *wsp*, *coxA*, *fbpA*, *ftsZ*, *gatB*, and *hcpA*, were sequenced and compared across samples. Two prevalent *w*Di strains were identified across the samples, and screening of clone libraries revealed possible coinfection of *w*Di strains in specific populations. *D. citri* mitochondrial cytochrome oxidase subunit I gene (mtCOI) were more divergent between *D. citri* populations that were infected with different *w*Di strains or had different infection statuses (single infection vs. coinfection). While we could not eliminate the possibility that maternal transmission may contribute to such patterns, it is also possible that *w*Di may induce cytoplasmic incompatibility in their host. These findings should contribute to the understanding of *w*Di population ecology, which may facilitate manipulation of this endosymbiont for management of citrus greening disease worldwide.

## Introduction

Bacterial endosymbionts of insects have a diverse and profound influence on host biology (Moran & Telang, 1998; Werren *et al*., 2008; Engelstädter & Hurst, 2009; Feldhaar, 2011). The effects these bacteria have on their host include nutrient provision or recycling (Douglas, 1998; Sabree *et al*., 2009; Hosokawa *et al*., 2010), protecting the host against infective agents or natural enemies (Oliver *et al*., 2002; Scarborough *et al*., 2005; Kambris *et al*., 2009; Moreira *et al*., 2009), manipulation of host reproduction or sexuality (Rousset *et al*., 1992a,b; Hurst *et al*., 1999; Engelstädter & Hurst, 2009), and stress tolerance (Montllor *et al*., 2002; Feldhaar, 2011). These effects may be exploited to achieve applicative purposes, such as controlling pest population sizes or interfering with transmission of insect‐borne diseases (Brelsfoard *et al*., 2009; Hancock *et al*., 2011; Iturbe‐Ormaetxe *et al*., 2011).

*Diaphorina citri* Kuwayama (Hemiptera: Liviidae) is a phloem‐feeding pest of citrus. This hemipteran is considered one of the most economically important citrus pests worldwide due to its capability to transmit the putative bacterial causal agent of citrus greening, *Candidatus* Liberibacter asiaticus (*C*Las) (Halbert & Manjunath, 2004). *C*Las is acquired by *D. citri* during feeding. The bacterial cells can enter the digestive tract, invade the hemolymph and salivary gland, and are then transmitted to a healthy plant, thereby completing a propagative, persistent transmission process (Hogenhout *et al*., 2008; Hall *et al*., 2013). *D. citri* was initially identified in Taiwan, China in 1908 (Kuwayama, 1908), and is found throughout most citrus growing regions of Asia and the Americas. The first description of *D. citri* in the United States occurred in Palm Beach County Florida in 1998. Analyses of the cytochrome oxidase gene indicates that *D. citri* in Florida originated from southwestern Asia (Boykin *et al*., 2012). Current control strategies, including tree removal, and insecticide and antimicrobial applications have limited or variable efficacy (Michaud, 2004; Qureshi *et al*., 2009; Tiwari *et al*., 2011; Hall *et al*., 2013; Stansly *et al*., 2014). These facts and the continued spread of citrus greening suggest the need for additional methods to supplement existing integrated pest management strategies.

In recent years, mounting evidence suggests that insect endosymbionts could hold keys to new approaches for managing insect‐transmitted diseases. Of particular interest are several strains of the alpha‐proteobacteria *Wolbachia*, which provide their host protection against different types of infective agents and potentially reduce the transmission of insect‐borne pathogens/parasites under controlled conditions (Kambris *et al*., 2009; Moreira *et al*., 2009; Hancock *et al*., 2011). Moreover, in some insects, *Wolbachia* induce cytoplasmic incompatibility (CI), a phenomenon where mating between same‐species individuals with different *Wolbachia* strains or infection status fails to produce viable offspring (Yen & Barr, 1973; Engelstadter & Telschow, 2009); these CI‐inducing strains may also facilitate disease management by manipulating vector populations (Sinkins & Gould, 2006; Bourtzis, 2008; Brelsfoard *et al*., 2009). Like many insect species, *D. citri* is also infected with *Wolbachia* (designated *w*Di) (Subandiyah *et al*., 2000; Guidolin & Consoli, 2013; Dossi *et al*., 2014; Hoffmann *et al*., 2014). Although the direct influence of *w*Di on *D. citri* biology remains to be determined, recent studies indicated that the relative abundance of *w*Di may be associated with the abundance of *C*Las within hosts (Fagen *et al*., 2012), and that *w*Di may contribute to the regulation of phage lytic cycle genes in *C*Las (Jain *et al*., 2017). These findings highlight the potential importance of *w*Di in the citrus greening disease system, and call for better characterization of *w*Di biology in general.

A fundamental step for understanding endosymbionts like *w*Di is to determine the distribution and diversity of the endosymbiont across different host populations. In this regard, several studies have investigated the genetic diversity of *w*Di across different populations (Saha *et al*., 2012; Guidolin & Consoli, 2013; Lashkari *et al*., 2014). Lashkari *et al*. (2014) found that the *w*Di *wsp* sequence was associated with host genetic diversity and hypothesized that *w*Di may induce CI in their host (Lashkari *et al*., 2014). In general, previous studies were based on sequences of only one or two *w*Di genes (*wsp* and *ftsZ*) (Saha *et al*., 2012; Lashkari *et al*., 2014), or restricted to populations in specific regions of the world (Guidolin & Consoli, 2013). Considering *D. citri*’s impact on citrus production worldwide and the mosaic nature of some *Wolbachia* genes, including *wsp* (Baldo *et al*., 2005), a broader and more robust assessment of *w*Di genetic diversity and its association with *D. citri* is necessary towards understanding the global diversity of *D. citri* populations.

In this study, we investigated genetic variation of *w*Di among 24 *D. citri* populations located across multiple countries. Six *Wolbachia* genes, including the surface protein gene (*wsp*) and genes used in the multilocus sequence typing (MLST) procedure (Baldo *et al*., 2006), *coxA*, *fbpA*, *ftsZ*, *gatB*, and *hcpA*, were sequenced for every sample to allow sensitive and robust analyses of *w*Di diversity. The MLST procedure recognizes different sequences of the same gene as separate alleles and unambiguously characterizes each bacterial strain based on the allele profiles of the genes analyzed (Baldo *et al*., 2006). Sequencing of *w*Di genes and subsequent clone library screening allowed identification of prevalent *w*Di profiles and the possible occurrence of *Wolbachia* coinfection among the *D. citri* populations tested. We also tested whether there is an association between *D. citri* and *w*Di’s genetic diversity by sequencing a mitochondrial cytochrome oxidase subunit I gene (mtCOI) fragment of *D. citri*. Obtaining the *w*Di and mtCOI from the exact same samples (instead of using data from other sources/individuals) helped assure that the associations between *w*Di and *D. citri* sequences drawn from the data were reliable. Results from this work could improve our understanding of *w*Di and *D. citri* ecology.

## Materials and methods

### Diaphorina citri DNA samples

Adult *D. citri* were sampled from Homestead and Fort Pierce, FL. Insects were also collected from two laboratory colonies; one derived from a Weslaco, TX population and the other from a (Oahu, HI) population (Table 1). Samples were collected from commercial and unmanaged citrus groves during 2015–2016. Whole‐body genomic DNA was extracted from individual insects using the DNeasy Blood & Tissue kit (Qiagen, Inc. Valencia, CA, USA) following the manufacturer's instructions. In addition, *D. citri* DNA was obtained from specimens stored at the United States Department of Agriculture, Animal and Plant Health Inspection Service, Center for Plant Health Science and Technology, Mission Laboratory; these included DNA isolates of *D. citri* collected from eleven countries and two U.S. territories during 2015 and 2016 (Table 1). DNA samples from a previous study of Florida *D. citri* populations (Chu *et al*., 2016) were also included in the analyses. Overall, 61 samples collected from 24 populations were used in the analyses.

**Table 1:** Details of *Diaphorina citri* samples used in this study

Sampling location	Number of samples	Source of sample
USA, FL, Clermont	4	Chu *et al*., 2016
USA, FL, Lake Alfred	5	Chu *et al*., 2016
USA, FL, LaBelle	5	Chu *et al*., 2016
USA, FL, Fort Pierce	6	This work (2016)
USA, FL, Homestead	6	This work (2016)
USA, HI, Oahu	1	Laboratory colony (2016)
USA, TX, Weslaco	1	Laboratory colony (2016)
USA, TX, Edinburg	1	The Mission Laboratory (2016)
American Samoa, Tafuna	1	The Mission Laboratory (2015)
Puerto Rico, Santa Isabel	2	The Mission Laboratory (2015)
Mexico, Tamaulipas, Cuidad Victoria	2	The Mission Laboratory (2015)
Mexico, Veracruz, Cazones	2	The Mission Laboratory (2015)
Trinidad and Tobago, Trinidad	2	The Mission Laboratory (2015)
Barbados, Golden Grove	3	The Mission Laboratory (2015)
Belize, Stann Creek	3	The Mission Laboratory (2015)
Colombia, Tolima, Armero‐Guayabal	2	The Mission Laboratory (2015)
Pakistan, Punjab, Multan	1	The Mission Laboratory(2015)
Pakistan, Punjab, Lalian	2	The Mission Laboratory (2015)
Thailand, Nakhon Si Thammarat, Meuang Nakhon Si Thammarat	2	The Mission Laboratory (2015)
China, Yunnan, Ruili	1	The Mission Laboratory (2015)
China, Fujian, Fuzhou	3	The Mission Laboratory (2015)
Singapore, Chinese Garden	2	The Mission Laboratory (2015)
Argentina, Salta, Yuchán,	2	The Mission Laboratory (2015)
Argentina, Jujuy, Fraile Pintado	2	The Mission Laboratory (2015)

### Amplification and sequencing of Wolbachia and D. citri mitochondrial genes

Fragments of seven genes were sequenced for all of the DNA isolates. These include *D. citri* mtCOI and six *Wolbachia* genes (*wsp*, *coxA*, *fbpA*, *ftsZ*, *gatB*, and *hcpA*). Amplifications of the *Wolbachia* genes and the *D. citri* mtCOI gene were conducted using primer pairs and PCR conditions described in previous studies (Baldo *et al*., 2006; Boykin *et al*., 2012) with slight modification (Table 2). GoTaq Colorless Master Mix (Promega Inc., Madison, WI, USA) was used for these assays. The final concentrations of the MLST and *wsp* primer pairs were 1 μmmol/L and the final volume was 40 μL for all of the reactions. The PCR products were purified using ExoSAP‐IT (USP Corp., Cleveland, OH, USA) and sequenced (from both forward and reverse ends) at the Interdisciplinary Center for Biotechnology Research at the University of Florida (ICBR).

**Table 2:** Details of primers used in this study

Target gene	Primer sequences (5′→3′)	Amplicon size	Annealing temperature	Reference
*gatB*	Forward: GAKTTAAAYCGYGCAGGBGTT Reverse: TGGYAAYTCRGGYAAAGATGA	471 bp	54 °C	Baldo *et al*., 2006
*coxA*	Forward: TTGGRGCRATYAACTTTATAG Reverse: CTAAAGACTTTKACRCCAGT	487 bp	54 °C	Baldo *et al*., 2006
*hcpA*	Forward: GAAATARCAGTTGCTGCAAA Reverse: GAAAGTYRAGCAAGYTCTG	515 bp	54 °C	Baldo *et al*., 2006
*ftsZ*	Forward: ATYATGGARCATATAAARGATAG Reverse: TCRAGYAATGGATTRGATAT	524 bp	52 °C	Baldo *et al*., 2006
*fbpA*	Forward: GCTGCTCCRCTTGGYWTGAT Reverse: CCRCCAGARAAAAYYACTATTC	509 bp	58 °C	Baldo *et al*., 2006
*wsp*	Forward: GTCCAATARSTGATGARGAAAC Reverse: CYGCACCAAYAGYRCTRTAAA	603 bp	58 °C	Baldo *et al*., 2006
*D. citri*’s mtCOI	Forward: AGGAGGTGGAGACCCAATCT Reverse: TCAATTGGGGGAGAGTTTTG	821 bp	53 °C	Boykin *et al*., 2012

### Data processing and analyses

All Sanger sequencing data were analyzed using Geneious 8.1.6 (Kearse *et al*., 2012). For each gene, sequence chromatograms were screened for errors or artifacts affecting sequence calls. Forward and reverse sequences were then assembled and the resulting sequences were compared among the samples in Geneious using MUSCLE (8 iterations) (Edgar, 2004). For *w*Di genes, unique sequences were searched against the *Wolbachia* MLST database (http://www.pubmlst.org/wolbachia/) (Baldo *et al*., 2006). The sequencing chromatograms of some amplicons in some samples exhibited dual peaks at several sequence positions. Dual chromatogram peaks at individual sequence positions were consistent among sequencing reactions with multiple alleles among the amplicon pool. As this is suggestive of cooccurrence of more than one *w*Di profiles within the same *D. citri* individual, forward and reverse chromatograms were compared to evaluate the consistency of these patterns. Individuals with strong evidence of coinfection in both forward and reverse chromatograms were grouped into “coinfection groups.” That is, “coinfection groups” were composed of those individuals that exhibited dual peaks of the same nucleotides at the same sequence locations for the same genes. PCR amplicons from genes of representative individuals of different coinfection groups were cloned and libraries were sequenced to verify coinfection and identify haplotypes.

### Construction and sequencing of clone libraries

To determine whether the coinfection patterns observed within some samples (described later in detail) were due to the infection of more than one *w*Di strains, representative DNA samples exhibiting the observed patterns were cloned and sequenced for each “coinfection group.” For each locus that exhibited strong signs of coinfection, two replicate libraries derived from independent PCR reactions of the same representative DNA sample were screened; between 42 and 47 clones were sequenced per library. A total of eight libraries (357 clones) were included in the analyses. Briefly, PCR reactions were conducted using the FideliTaq PCR Master Mix (with proofreading; Affymetrix, Santa Clara, CA, USA) and the PCR conditions/protocols described above. The PCR products were purified with the QIAquick PCR Purification Kit (Qiagen, Inc.) and cloned into the pGEM‐T Easy Vector (Promega, Inc.). Transformed clones (*Escherichia coli* JM109) carrying plasmids with the inserts were submitted for Sanger sequencing at ICBR. To differentiate putative *w*Di alleles from random errors introduced during PCR reactions, sequences that were present in two independent libraries (prepared using independent PCR reactions) and explained the original coinfection pattern were identified. These sequences represent alleles that were sufficiently abundant to be repeatedly detected within the sequencing depth used in this work, which should also be free of random PCR or sequencing errors.

### Bayesian analysis of D. citri mtCOI sequences

Assembly of mtCOI sequences was conducted using Geneious; low‐quality ends were trimmed (Error Probability Limit = 0.05). The sequences were then curated and aligned. A 752 bp fragment available for all 61 samples was then compared among each other. Unique mtCOI sequences were subjected to Bayesian analyses (Huelsenbeck & Ronquist, 2001), using a *Cacopsylla coccinea* mtCOI sequence (Que *et al*., 2016) as an outgroup. Model selection and data analyses were conducted using MEGA 7 (Kumar *et al*., 2016) and the MrBayes plugin for Geneious, respectively. The available model that had the lowest Bayesian Information Criterion (BIC) score was selected (HYK85) (Hasegawa *et al*., 1985). The Markov chain Monte Carlo settings were: four heated chains, heated chain temperature = 0.2, chain length = 1 100 000, subsampling frequency = 200, burn‐in length = 100 000. The priors were set as the default parameters. The average standard deviation of split frequencies was below 0.01 at the end of the analysis.

## Results

### Identification of two prevalent wDi profiles

**Figure 1:**
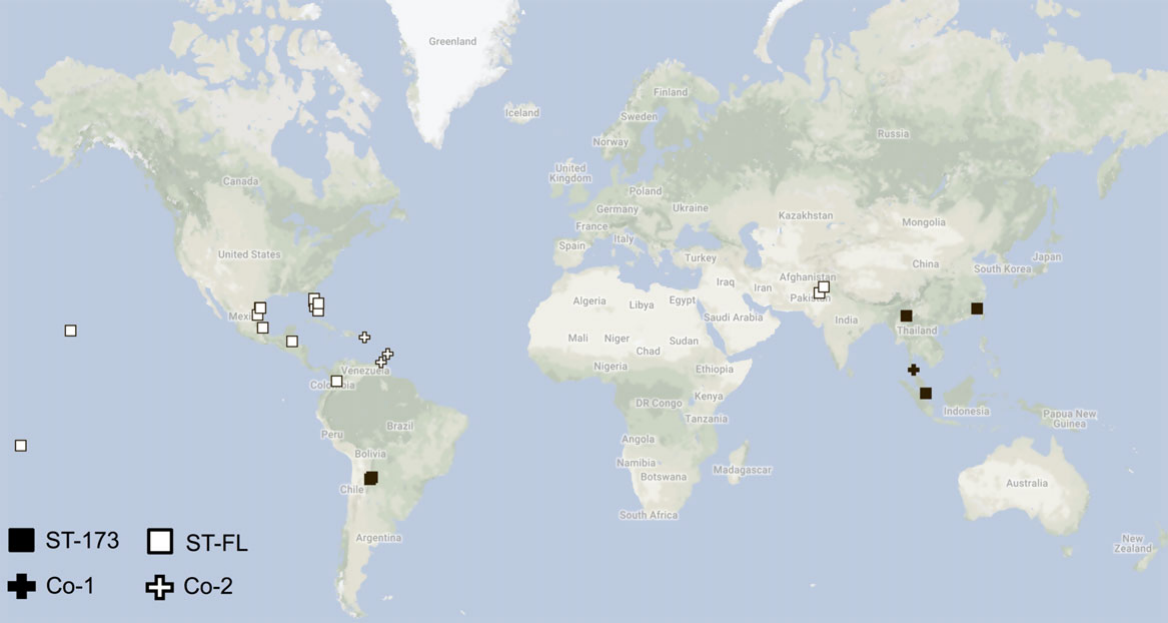
Distribution of *Diaphorina citri* populations with different *Wolbachia* (*w*Di) infection profiles. Symbols representing each infection profile are illustrated on the lower‐left. Created using Google Maps.

*Wolbachia* was detected in all 61 DNA samples tested. Compilation of the sequencing data revealed that in 20 of the 24 populations surveyed, all individuals exhibited one of two *w*Di profiles. Psyllids with each profile appear to be infected by a single dominant *w*Di strain. One *w*Di profile includes all five MLST alleles completely matching ST‐173 in the MLST database (the database assigns different strains with different strain numbers); this profile was detected in all individuals sampled from China, Singapore, and Argentina. A different profile was detected in all samples originated from the United States (Florida, Hawaii, Texas), American Samoa, Belize, Mexico, Pakistan, and Colombia; the alleles of this profile did not completely match to any known profiles in the MLST database, but were identical to sequences of a published *Wolbachia* genomic assembly obtained from Florida‐reared *D. citri* (Saha *et al*., 2012); this profile is hereafter referred to as ST‐FL (Table 3; Fig. 1). Overall, only the Thailand, Trinidad, Barbados, and Puerto Rico populations had samples exhibiting *w*Di profiles different from ST‐173 and ST‐FL.

**Table 3:** *w*Di allelic profiles identified in this study

	*Wolbachia* genes							
*w*Di profile	*gatB*	*coxA*	*hcpA*	*ftsZ*	*fbpA*	*wsp*	Location	Number of samples
ST‐173	109†	86	29	81	27	160	China, Singapore, Argentina	10
Co‐1	*109/106/Co‐1a* ‡	86	29	*81/7/208*	27	160	Thailand	2
ST‐FL	246	11	101	209	4	308	USA, Mexico, Belize, American Samoa, Pakistan, Colombia	42
Co‐2	246	11	*101/106/Co‐2a* ‡	*209/208*	4	308	Puerto Rico, Trinidad, Barbados	7

^†^For sequences matching (100%) the alleles in the Multilocus Sequence Typing (MLST) database, the allele identifiers (IDs/numbers) are shown.

^‡^Alleles detected via clone library analyses are italicized. Among them, Co‐1a and Co‐2a did not have exact matches in the MLST database.

While the MLST method examines genetic diversity by looking at unique allelic profiles, comparing allele sequences from this work with those of previous studies could help understand how ST‐173 and ST‐FL fit within the known *Wolbachia* diversity. In an earlier work (Saha *et al*., 2012), phylogenetic analyses were conducted using *Wolbachia* sequences of various insect species and *D. citri* populations from Florida, USA and China. There, it was shown that all *w*Di tested belonged to *Wolbachia* supergroup B, and that based on the *wsp* sequences, the Floridian *w*Di isolate belonged to a sub‐clade of supergroup B, which is distinct from Chinese isolates (samples from Beihai, Liuzhou, Fuzhou, and Shenzhen, China). Analysis of the *wsp* sequences from the present work indicated that sequences of ST‐173 and ST‐FL matched 100% to sequences of *D. citri* from Beihai, China (Accession number: GQ385974.1) and Florida, USA (Genome assembly accession number: PRJNA29451), respectively.

### Coinfection patterns detected in specific D. citri populations

Variability in the *gatB* and *ftsZ* sequences was detected between the two Thailand samples tested. One sample's *gatB* and *ftsZ* sequences matched completely to the alleles of ST‐173, while those of the other do not. Further inspection of their chromatograms revealed that such inconsistencies were due to additional “peaks” (i.e., signals of florescence from alternative nucleotides) present at several nucleotide sites, which resulted in different sequence calls. These peaks were found in both Thailand samples at the same nucleotide sites. Similar intrapopulation variations in the *w*Di profile were also detected in sequencing data of *D. citri* originated from the West Indies (Trinidad, Barbados, and Puerto Rico); the *ftsZ* sequences varied within the Puerto Rican and Trinidadian populations, while variation in the *hcpA* sequence was only detected within the Puerto Rican population. A comparison of the *ftsZ* and *hcpA* chromatograms from these samples and the Barbados samples revealed that the presence of additional peaks in several nucleotide sites led to inconsistent or different sequence calls; these peaks, albeit differed in their relative sizes, were detectable in the *ftsZ* and *hcpA* chromatograms of all samples collected from the West Indies.

**Figure 2:**
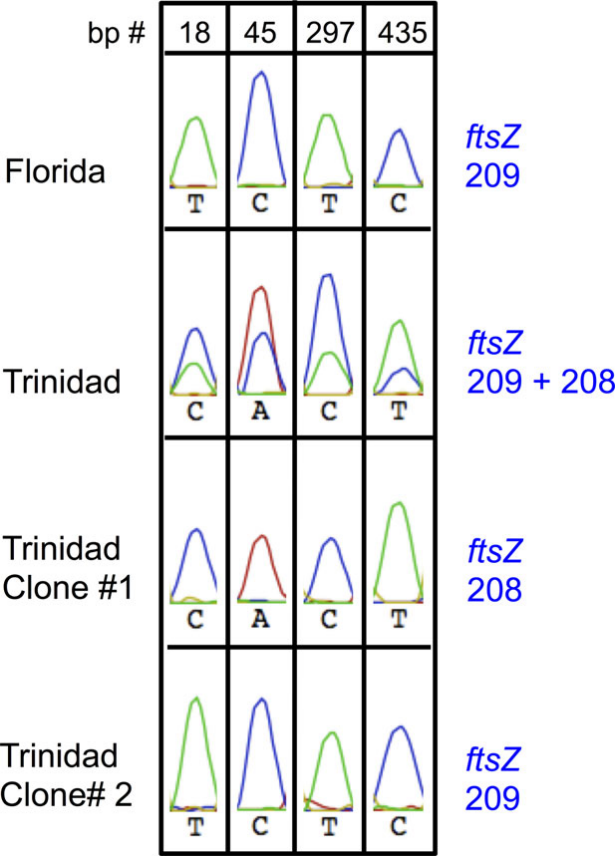
Examples of sequencing chromatograms illustrating strong *Wolbachia* “coinfection patterns” detected in this study and how clone library sequencing was used to validate these observations. The origins of the samples and the corresponding allele numbers (based on the multilocus sequence typing [MLST] database) are shown on the left and right, respectively. Strong coinfection patterns can be detected at various nucleotides of specific gene sequences (in this case at bp 18, 45, 297, and 435 of a *ftsZ* fragment). A sample originated from Lake Alfred, Florida only carries allele 209, while a coinfected sample collected from Trinidad contains more than one allele. Screening of clone libraries constructed from the Trinidad sample identified clones carrying different alleles (alleles 208 and 209; lower two panels) and explained the patterns seen in the original chromatogram. The peaks shown are parts of the actual chromatograms of the sequencing data (in the Geneious software). Peaks with different colors represent different nucleotides. Red: A; Green: T; Blue: C.

An example illustrating strong “coinfection patterns” described above is shown in Figure 2. The substantial sizes of the additional peaks and their presence in multiple samples/populations indicated that they are not merely artifacts of PCR or experimental errors. Based on these patterns, the *w*Di profiles of samples collected from the Thailand and West Indies were sorted into two coinfection profiles/groups, designated as “Co‐1” and “Co‐2,” respectively (Fig. 1).

Notably, although presence of additional peak signals in Co‐1 and Co‐2 samples resulted in sequence calls different from those of the ST‐173 and ST‐FL alleles, signals matching the ST‐173 or ST‐FL sequences were detected in the Co‐1 and Co‐2 samples, respectively; these observations were further validated using clone library screening (described later in detail). To determine whether the additional peaks in Co‐1 and Co‐2 chromatograms (peaks different from those of ST‐173 or ST‐FL alleles) were unique to each coinfection group, signals identified in Co‐1 (*gatB* and *ftsZ*) and Co‐2 (*ftsZ* and *hcpA*) samples were manually searched against ST‐173 and ST‐FL individuals/chromatograms, respectively. The additional peaks visible in the West Indies samples were not found in ST‐FL populations (Fig. S1). There were minor signals in some ST‐173 samples, particularly those collected from China and Singapore, that resembled the additional peaks found in the *gatB* and *ftsZ* data of Co‐1 samples. Nevertheless, the relative abundance of such signals was weaker than those found in the Co‐1 samples and in no cases did they result in sequence calls different from those of the ST‐173 profile. Thus, although discrepancy between Co‐1 and ST‐173 populations should be interpreted with caution, this study grouped them as different *w*Di profiles.

### Sequencing and analyses of clone libraries

Using representative DNA samples of the Co‐1 and Co‐2 groups, we constructed and screened clone libraries of genes whose chromatogram exhibited strong patterns of coinfection (one representative DNA sample for each “gene × group”). For the Co‐1 group, libraries of *ftsZ* and *gatB* were screened (using a Thailand sample); for Co‐2, we analyzed libraries of *ftsZ* and *hcpA* (using a Trinidad sample and a Barbados sample, respectively). An example illustrating how clone screening was used to validate strong coinfection patterns detected in the sequencing data is shown in Figure 2. When aligning clone sequences to the original chromatographs of their respective *D. citri* DNA sample, the combinations of sequences from different alleles (of the same gene) reflected the coinfection patterns observed in the original data. Data from the clone library analyses showed that Co‐1 and Co‐2 samples indeed harbor more than one *w*Di strains; Co‐1 and Co‐2 populations not only have alleles completely matching the ST‐173 and ST‐FL profiles, respectively, but also carry additional alleles not found in the two prevalent *w*Di strains (Table 3). Most of the additional alleles have exact matches in the MLST database; the sequences of two alleles that did not have matches in the MLST and GenBank databases have been deposited into GenBank (accession numbers: KX990271 and KX990272). Comparisons of these sequences with other *w*Di alleles detected are shown in Figures S2–S4. The fact that these additional alleles do not match to any of the ST‐173 and ST‐FL alleles shows that the coinfection patterns observed did not result from cooccurrence between ST‐173 and ST‐FL samples.

### Association between mtCOI sequences and wDi profiles

**Figure 3:**
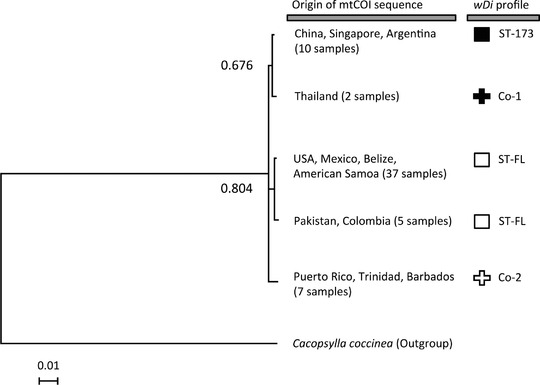
Bayesian analysis of mtCOI sequences of *D. citri* samples with different *Wolbachia* (*w*Di) infection profiles. The *w*Di profiles for the samples are shown on the right. The sequence of *Cacopsylla coccinea*’s mtCOI was used as an outgroup. The posterior probabilities are shown next to the branches.

Collections with the ST‐173 profile all shared the same mtCOI sequence (Fig. 3). With the exception of the Pakistani and Colombian samples, *D. citri* carrying ST‐FL all shared an mtCOI sequence different from that of ST‐173 samples (Fig. 3). The Pakistani and Colombian samples shared another mtCOI sequence, which had one nucleotide different from that of the other ST‐FL populations (Table S1).

The Co‐1 and Co‐2 populations’ mtCOI sequences were different from those of the ST‐173 and ST‐FL populations; the Co‐1 and Co‐2 mtCOI sequences were also different from each other (Fig. 3). Among all *D. citri* that carried the ST‐FL alleles (ST‐FL and Co‐1 populations), *D. citri* from the West Indies had the most different mtCOI sequences compared to the other *D. citri* (five to six nucleotide differences), while the other ST‐FL *D. citri* only had up to one nucleotide difference among each other (Table S1). Most of the additional alleles have exact matches in the MLST database, and their sequences are available at https://pubmlst.org; the sequences of two alleles that did not have matches in the MLST and GenBank databases were deposited into GenBank (accession numbers: KX990271 and KX990272).

## Discussion

Two prevalent *w*Di strains, ST‐173 and ST‐FL, were identified in this study. The distribution of each *w*Di strain was not entirely restricted to specific geographic regions, suggesting that factors such as human transportation may have played a major role in distributing *D. citri* (along with their *w*Di strains) to various parts of the world. Overall, the distribution patterns of profiles ST‐173 and ST‐FL also corroborated findings from previous works. For example, ST‐173 was reported as the most prevalent strain infecting *D. citri* in many populations of Brazil (Guidolin & Consoli, 2013), an area located closely to the ST‐173‐carrying Argentinean populations studied in this work. Previously, Saha *et al*. (2012) showed that *w*Di in Floridian *D. citri* populations belonged to a supergroup B sub‐clade different from Chinese isolates, and suggested that *D. citri* in Florida did not originate from China. Here, the data also showed clear distinction between the Florida (ST‐FL) and Chinese (ST‐173) *w*Di allelic profiles, and that the ST‐173 and ST‐FL sequences were identical to those of the Florida, USA and a Chinese samples (from Beihai, China), respectively. Therefore, the data obtained from analysis of six *w*Di genes not only supported previous findings, but also provided more robust support for the global distribution, diversity, and prevalence of major *w*Di strains as compared with data from analysis of *wsp* and *ftsZ* alone.

Coinfection of more than one dominant *w*Di strains was detected in populations located in Thailand (Co‐1) and the West Indies (Co‐2). Interestingly, the *Wolbachia* strains that the Co‐1 and Co‐2 populations carry included ST‐173 and ST‐FL, respectively. Since Thailand is located near China and Singapore (ST‐173 areas), and the Co‐2 populations are distributed near ST‐FL populations, it is plausible that *D. citri* in Co‐1 and Co‐2 populations may have originated from their surrounding areas. Psyllids in the coinfected populations may have acquired additional *w*Di strains as they moved into their current locations, or have experienced changes in host‐microbe interactions as a result of altered environments. Arthropods are known to acquire different *Wolbachia* strains horizontally via brief blood/wound contacts with insects sharing same ecological niches (Rigaud & Juchault, 1995) or obtain exogenous strains during attack by predators and parasitoids (Werren *et al*., 1995). Environmental factors, host genetics, or infection by other microbial species may also contribute to the infection densities of *Wolbachia* strains (Goto *et al*., 2006; Mouton *et al*., 2006; Chu *et al*., 2016). It is possible that such interactions or factors may have occurred in some of the coinfected populations. Further investigation is needed to determine the sources and cause of the coinfection patterns observed in this work.

Examination of mtCOI sequences revealed geographic patterns of *D. citri* and *w*Di populations that are in agreement with those of other studies. Comparison among mtCOI genes suggests two separate introductions of *D. citri* into the Americas, one in North America and the other in South America (de León *et al*., 2011), while our data showed that the mtCOI sequences in these two areas are relatively different. The difference between mtCOI sequences from North America and Southeast Asia identified in our study was also concordant with patterns shown in other studies (Lashkari *et al*., 2014).

Similar to a previous study focusing on mtCOI and *w*Di *wsp* alone (Lashkari *et al*., 2014), the current analyses including multiple *w*Di genes showed that populations carrying different *w*Di strains have different mtCOI, and that samples with different haplotypes do not cooccur within the same population. Moreover, our data showed that the Co‐2 populations are coinfected with strains that were undetected in all of the ST‐FL populations, and that these *D. citri* had the most different mtCOI sequence compared to the haplotypes of ST‐FL populations (Table S1). Specifically, the geographically distant North American, Hawaiian and Belizean populations share the same ST‐FL profile, yet Co‐2 (West Indies) populations have the most divergent mtCOI sequence as compared with those from all other ST‐FL‐carrying populations (including populations located in nearby countries such as the United States, Belize, Mexico, and Colombia; Table S1). In some insect species, infection with different *w*Di strains in males and females could induce bidirectional CI, a phenomenon that may facilitate genetic divergence (Teschlow *et al*., 2002), while coinfection with additional *Wolbachia* strains could have an additional effect, such that crosses between coinfected and singly infected insects may result in unidirectional CI (Sinkins *et al*., 1995; Dobson *et al*., 2001). Therefore, while we could not rule out the possibility that maternal transmission may contribute to patterns detected in this work, it is also possible that infection with different *w*Di strains or the difference in infection status may have induced CI and facilitated genetic divergence (Sinkins *et al*., 1995; Dobson *et al*., 2001; Telschow *et al*., 2002). And thus, although difficulties in obtaining larger sample numbers, DNA quantities, and live insects across different geographic locations restricted this study from fully validating the potential links between CI and the patterns observed, the data presented here does warrant further investigation of *w*Di's effect on *D. citri* reproduction.

In the present work, samples within same populations all shared identical *w*Di profiles. However, it is likely that other less prevalent *w*Di profiles (infection status) or mtCOI haplotypes may also exist within populations. A previous study of Brazilian *D. citri* populations found that, although the majority of individuals tested carried the same *w*Di strain (ST‐173), some individuals within the same populations could harbor different *w*Di strains (Guidolin & Consoli, 2013). Our previous study across Florida *D. citri* populations also indicated that a very small proportion of *D. citri* individuals could be free of *w*Di infection (Chu *et al*., 2016), suggesting that intrapopulation variation in *w*Di profiles could exist in other populations as well. Nevertheless, given our repeated detection of identical *w*Di profiles among multiple samples of the same or closely located populations, the data was able to provide a broad assessment of the distribution patterns of prominent *w*Di strains across continents. It is also important to note that the approaches used in this work were not intended for detection of *Wolbachia* strains/alleles that have lower infection densities/abundances. Previous findings indicate that *Wolbachia* functions are dependent on infection levels (Breeuwer & Werren, 1993; Hurst *et al*., 2000; Unckless *et al*., 2009); therefore, *w*Di strains with higher within‐host density may be more likely to have biologically relevant effects on the host. Thus, the methods used in this work could facilitate detection of additional strains that may have substantial influence on *D. citri* biology, such as CI induction.

Characterizing the diversity and ecology of *w*Di could help elucidate the population structures of *D. citri* and *w*Di as well as their interactions in the field. In strategies used to control insect pests or insect‐borne pathogens, *Wolbachia*‐induced CI could also play an important role in reducing insect population size or acting as a drive system for disseminating desirable genes/alleles (Sinkins & Gould, 2006; Bourtzis, 2008). This study identified prevalent *w*Di strains infecting *D. citri* across widespread populations, *w*Di coinfection in field populations, and interesting associations between *w*Di infection status and *D. citri* mtCOI sequences. Findings from this work may facilitate the understanding of *w*Di–*D. citri* interactions that could benefit the development of control strategies for *D. citri* and citrus greening. These data also show that transportation of an insect pest could enable dissemination of different endosymbiont strains across the globe, potentially resulting in complex and diverse host–microbe associations in the field.

## Disclosure

The authors declare that they have no conflict of interest.

## Supporting information


**Table S1**. Differences among mtCOI sequences of *D. citri* with different *w*Di profiles.
**Fig. S1**. Examples of minor signals in ST‐173 samples that resembled the additional peaks found in Co‐1 samples. The peaks shown are parts of the chromatograms of the sequencing data (in the Geneious software). Peaks with different colors represent different nucleotides. Red: A; Green: T; Blue: C; Yellow: G.
**Fig. S2**. Alignment of *gatB* sequences detected in this study.
**Fig. S3**. Alignment of *ftsZ* sequences detected in this study.
**Fig. S4**. Alignment of *hcpA* sequences detected in this study.
