# Supplementary material for: Genetic variation and potential coinfection of Wolbachia among widespread Asian citrus psyllid (Diaphorina citri Kuwayama) populations
Source: Insect Sci. 2018 Feb 13;26(4):671–82. doi: 10.1111/1744-7917.12566 (PMC7379232; doi:10.1111/1744-7917.12566)
Supplement: Supplementary file 1 — Table S1. Differences among mtCOI sequences of D. citri with different wDi profiles. Fig. S1. Examples of minor signals in ST‐173 samples that resembled the additional peaks found in Co‐1 samples. The peaks shown are parts of the chromatograms of the sequencing data (in the Geneious software). Peaks with different colors represent different nucleotides. Red: A; Green: T; Blue: C; Yellow: G. Fig. S2. Alignment of gatB sequences detected in this study. Fig. S3. Alignment of ftsZ sequences detected in this study. Fig. S4. Alignment of hcpA sequences detected in this study. [file INS-26-671-s001.docx]

**Supporting information**

**Methods**

**Amplification of *Wolbachia* and *D. citri* mitochondrial genes**

Polymerase chain reactions (PCR) targeting the *Wolbachia* genes and *D. citri*’s mtCOI gene were carried out using the GoTaq Colorless Master Mix (Promega Inc., Madison, WI). Among the DNA samples tested, some were obtained from a collection at the United States Department of Agriculture, Animal and Plant Health Inspection Service, Center for Plant Health Science and Technology, Mission Laboratory. To assure that the samples were not degraded during storage or shipping and to avoid false negatives during detection/sequencing of *w*Di genes, polymerase chain reactions (PCR) targeting the mtCOI sequence (together with those of the newly extracted samples) was first carried out using the following conditions: 95°C for 2 min, followed by 35 cycles of 95°C for 30 s, 53°C for 30 s, 72°C for 1min, and a final extension at 72°C for 10 min. The final concentration for mtCOI primers was 0.4 μM and the reaction volume for each sample was 40 μl. For each reaction, one microliter of the DNA extract (10 ng for newly extracted samples) was used as the template. After confirming the presence of the mtCOI amplicon in the samples, amplification of *w*Di’s MLST (multilocus sequence typing) and *wsp* genes was then conducted; the PCR conditions for these reactions were: 95°C for 2 min, followed by 37 cycles of 95°C for 30 s, annealing for 45 s, 72°C for 1 min, and a final 10 min extension at 72°C. The annealing temperatures for different primer pairs used are shown in Table 2. The final concentrations for the primers were 1 μM and the reaction volume for these assays was 40 μl. After PCR, gel electrophoresis was used to confirm that no amplicons were produced in the non-template controls.

| **Table S1.** Differences among mtCOI sequences of *D. citri* with different *w*Di profiles. | | | | | |
| --- | --- | --- | --- | --- | --- |
|  | ST-FL-P^*^ | ST-FL | ST-173 | Co-1 | Co-2 |
| ST-FL-P^*^ | 0^**^ | 1 | 4 | 5 | 6 |
| ST-FL | 1 | 0 | 3 | 4 | 5 |
| ST-173 | 4 | 3 | 0 | 1 | 4 |
| Co-1 | 5 | 4 | 1 | 0 | 5 |
| Co-2 | 6 | 5 | 4 | 5 | 0 |
| *Name of *w*Di profiles. The *w*Di profile of Pakistani *D. citri* is designated as ST-FL-P; the profile of other populations carrying ST-FL is designated as ST-FL. | | | | | |
| **Number of nucleotide differences between each pair of mtCOI alleles (within the 752 bp alignment). | | | | | |

**Fig. S1.** Examples of minor signals in ST-173 samples that resembled the additional peaks found in Co-1 samples. The peaks shown are parts of the chromatograms of the sequencing data (in the Geneious software). Peaks with different colors represent different nucleotides. Red: A; Green: T; Blue: C; Yellow: G.

**
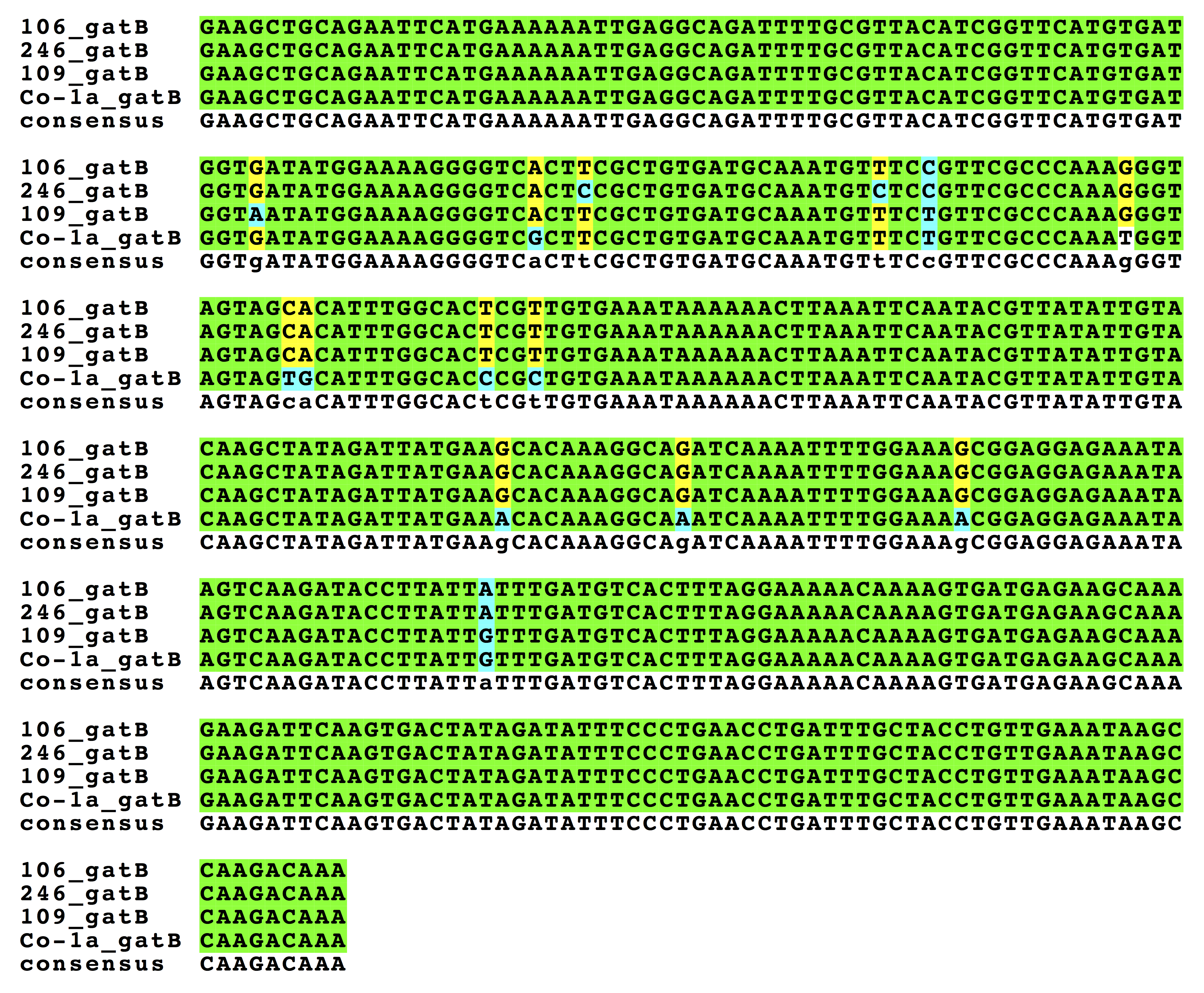
**

**Fig. S2.** Alignment of *gatB* sequences detected in this study.

**
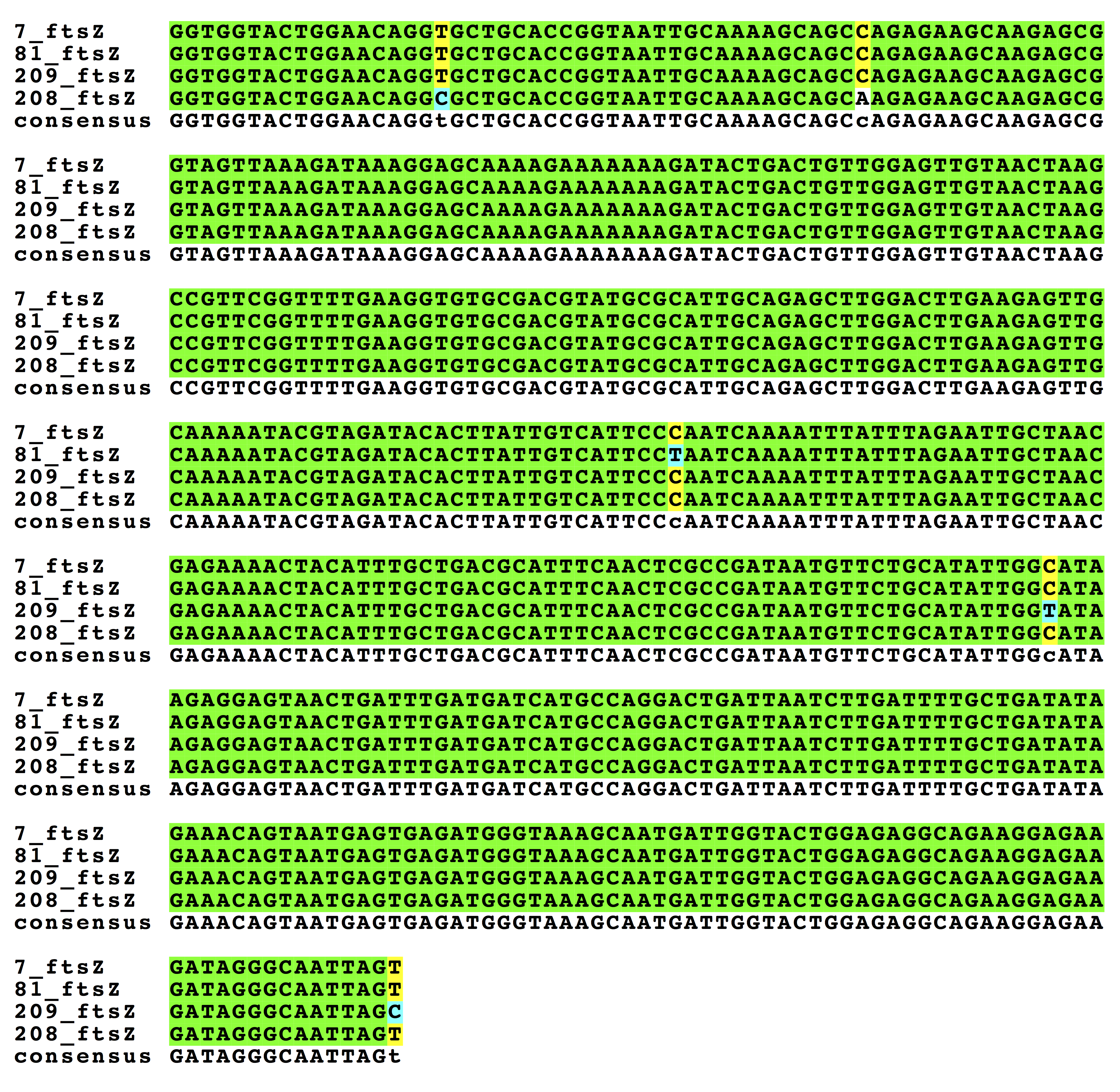
**

**Fig. S3.** Alignment of *ftsZ* sequences detected in this study.


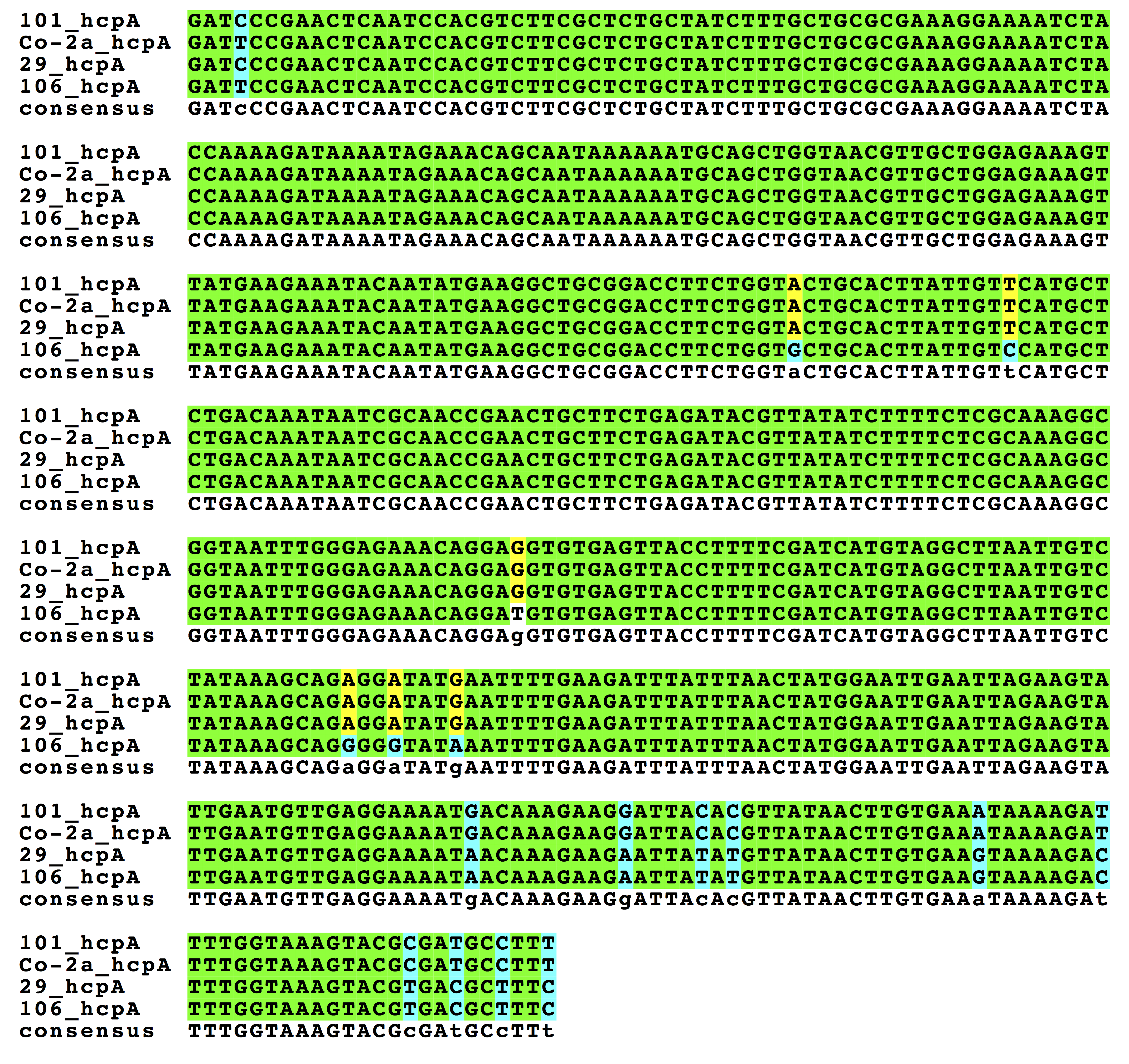


**Fig. S4.** Alignment of *hcpA* sequences detected in this study.
